# Demographics and clinical characteristics of alcohol-related admissions in a tertiary care hospital in Qatar: Does age matter?

**DOI:** 10.5339/qmj.2021.36

**Published:** 2021-09-09

**Authors:** Menatella Abdelnaby, Tasnim Abdalla, Hend Al-Kahtani, Dana Al-Rayashi, Rim Bashir, Yara Wanas, Ahmed Al-Neama, Hassan Ibrahim, Hussain Ibrahim, Aisha Al-Adab, Mohammad Asim, Ayman El-Menyar

**Affiliations:** ^1^College of Medicine, Qatar University, Doha, Qatar E-mail: aymanco65@yahoo.com; ^2^Department of Medicine, Hamad General Hospital, Doha, Qatar; ^3^Trauma Surgery Section, Department of Surgery, Hamad General Hospital, Doha, Qatar; ^4^Clinical Medicine, Weill Cornell Medical School, Doha, Qatar

**Keywords:** alcohol consumption, age distribution, blood alcohol concentration, ethanol, emergency department, Qatar

## Abstract

Background: Alcohol consumption is a major cause of acute and chronic health conditions associated with comorbidities and traumatic injuries, despite its partial prohibition in some countries. Moreover, alcohol-related hospital admissions increase the burden on the healthcare system. More than 80% of the population in Qatar comprises expatriates. This study aimed to analyze the demographics and clinical characteristics of subjects with alcohol-related emergency department (ED) visits/hospitalization with respect to different age groups in a single tertiary hospital in Qatar.

Methods: It is a retrospective observational study of adult patients who visited the ED at Hamad General Hospital between January 2013 and March 2015 and were screened positive for alcohol use. Collected data included sociodemographic characteristics, blood alcohol concentration (BAC), pattern of admission, previous medical history, laboratory investigations, treatment, hospital course, and mortality. Data were compared with respect to the distribution of age groups such as < 25, 25–34, 35–44, 45–54, and >55 years.

Results: In total, 1506 consecutively admitted patients screened positive for alcohol use were included in the study; the majority of them were males (95.6%), non-Qatari nationals (71.1%), and aged 35–44 years (30.9%). The age groups 35–44 years and 45–54 years showed the highest median BAC ([0.24 interquartile range (IQR: 0.14–0.33)] and [0.24 (IQR: 0.13–0.33)], respectively) as compared to the other age groups (P = 0.001). The pattern of hospital admission, sociodemographic status, presence of comorbidities, laboratory investigations, and mortality showed specific age-related distribution. Particularly, young adults were more likely to have a previous ED visit due to trauma, whereas older patients’ previous hospital admissions were mostly related to various underlying comorbidities.

Conclusion: This study highlighted the patterns of age and clinico-epidemiological status of patients with alcohol-attributable hospital admissions. Our study showed that alcohol consumption was higher among the working-age group. Further studies are needed to investigate changes in the alcohol consumption patterns that may help plan for allocation of health resources and prevention of alcohol-related problems.

## Introduction

Alcohol consumption is a public health crisis worldwide that is often associated with acute and chronic negative health consequences such as road traffic injuries, mental and behavioral disorders, hepatic cirrhosis, and cardiovascular diseases.^1,2^ Evidence from the Western world had suggested that alcohol-related visits to the emergency department (ED) and hospital admissions remarkably increase the healthcare burden in several developed countries.^3,4^ A previous study from Canada showed that the rate of alcohol-related hospital admissions was more frequent among males than females and the crude rates for alcohol-related hospitalizations peaked in the age groups of 55–59 years and 60–64 years.^5^ Moreover, the incidence of alcohol-related hospital visits is increasing in many developing countries, further necessitating appropriate screening and detection.^6^

However, in many Middle Eastern countries, alcohol abuse is a taboo that is partially regulated because of religious and cultural beliefs. Despite the cultural, religious, and legal constraints, the problem of alcohol consumption does exist in many Arabian Gulf countries.^7^ In Qatar, road traffic accidents are the leading cause of death, whereas overspeeding and driving under the influence of alcohol are associated with severe injuries or poor outcomes.^8,9^ El-Menyar et al., conducted a retrospective observational study between 2009 and 2012 to assess the patterns of alcohol screening in patients admitted at a national referral hospital.^10^ It was found that 38% were tested positive for blood alcohol concentration (BAC) among the screened patients; the majority of them were males (97%) with the mean age of 37.5 ± 11.6 years.

A higher proportion of young individuals are involved in heavy drinking episodes and alcohol consumption, which usually declines with age.^11^ However, little is known about the pattern of alcohol use among patients with different age groups admitted to the EDs in Qatar. Identifying the age patterns of drinking might help in informing the public health authorities to develop strategies for the prevention of alcohol abuse, control alcohol-related problems, and understand the trend of alcohol consumption in the population with special reference to specific age groups. This study aimed to analyze the socioeconomic characteristics, comorbidities, and alcohol-related hospitalization/ED visit patterns with respect to different age groups.

## Methods

It was a retrospective observational study of adult (age ≥ 18 years) patients screened positive for alcohol use upon presentation to the ED and/or were admitted to Hamad General Hospital (HGH) in Qatar between January 2013 and March 2015 because of various reasons. HGH is one of the Hamad Medical Corporation (HMC) facilities that offer free-of-charge treatment for all residents attending the ED. The selection criteria for alcohol screening were not clearly captured from the database. However, it was mostly based on the clinical suspicion and discretion of the treating physician as there was no standard guideline or mandate during the study period. The records of patients with negative alcohol screening and those without a recorded demographic data or under the age of 18 years were excluded. Data included socio-demographic characteristics (age, gender, nationality), employment, educational status, pattern of admission, comorbidities, drug history, laboratory investigations (liver and kidney function profiles), BAC, ethanol level, previous ED visit and hospital admission, admitting services, need for psychiatric consultation, and mortality. These data were retrieved from electronic medical records and patients’ charts. Medical and drug history included diabetes mellitus, hypertension, liver disease, coronary artery disease (CAD), cardiac arrest, arrhythmia, congestive heart failure, and the prescription of benzodiazepine and thiamine. One millimole of ethanol per liter of blood equals 4.61 milligrams of ethanol per 100 milliliters of blood. To convert the serum ethanol level to BAC, move the decimal point three places to the left. This observational study was approved by the Institutional Review Board at Hamad Medical Corporation (MRC15447/15) and Ethics Committee of Qatar University (QU-IRB 1098-E/19).

### Statistical analysis

Data were presented as proportions, medians, interquartile range, and means ± standard deviation as appropriate. Measured data were tested for normality using histograms, and the mean (SD) was reported, if the data were normally distributed. In case the distribution of data was skewed, the median and interquartile range (IQR) were reported. Descriptive statistics were used to evaluate the sociodemographic and clinical variables. Data were compared for the distribution of the study age groups such as < 25 years, 25–34 years, 35–44 years, 45–54 years, and >55 years. Based on these age groups, patients were also subgrouped into Qataris and non-Qataris. Differences in categorical variables between respective groups were analyzed using the chi-square test. The continuous variables were analyzed between the age groups using ANOVA for normally distributed data, whereas the non-parametric Kruskal–Wallis test was performed for continuous skewed data. Box-plots were used to compare the levels of ethanol and BAC among different age groups. A two-tailed P-value of < 0.05 was considered statistically significant. Data analysis was conducted using the Statistical Package for the Social Sciences, version 21 (SPSS, Inc, Chicago, IL).

## Results

During the study period, a total of 1506 cases of alcohol-related ED admissions/visits were recorded. Table 1 shows the demographic characteristics of the participants stratified according to different age groups. The majority of patients were males (95.6%), non-Qataris (71.1%), and were more likely to be within the age groups of 25–34 years (30.9%) and 35–44 years (28.4%). The mean age of the non-Qatari patients was significantly lower than the Qatari nationals (34.8 ± 10.9 vs. 43.9 ± 12.1; P < 0.001). The study age groups were comparable for gender and previous ED visit. Non-Qataris were more likely to be younger in age ( < 25 years and 25–34 years; P = 0.001), whereas a higher frequency of patients in the age groups of 45–54 years and ≥ 55 years were Qataris. Moreover, patients in the age group 45–54 years and ≥ 55 years had a significantly higher frequency of recurrent hospital admission (P = 0.001). Young patients ( < 25 years) with a history of previous hospital admission were more likely to be related to traumatic injuries. In contrast, patients with advanced age ( ≥ 55 years) were frequently admitted in the past for treatment not related to trauma (P = 0.001). Patients under the age of 25 years were primarily unmarried and unemployed compared with those in the age group of 35–44 years who were most likely to be employed and married (P = 0.001 for all).

Table 2 shows the comorbidities, admitting services, and mortality according to different age groups. Hypertension (27.2%) was the most common comorbidity followed by diabetes mellitus (22.6%) and liver diseases (13.0%). The frequency of hypertension (76.6%), diabetes mellitus (70.5%), liver dysfunction (42.6%), and CAD (48.5%) were significantly higher among older patients ( ≥ 55 years) than other age groups (P = 0.001 for all). Medication history showed that thiamine (57.1%) and benzodiazepine (25.7%) were the frequently used medications. The frequency of medication did not differ significantly among the study groups. Patients in the age groups of 35–44 years and 45–54 years frequently had an emergency short stay (P = 0.02). In contrast, younger patients mostly in the age group of < 25 years and 25–34 years had frequent admissions to the trauma resuscitation unit and trauma ICU (P = 0.001). Patients aged ≥ 55 years had highest proportion of admissions in the medical ICU (6.9%), cardiac ICU (3.0%), and cardiac ward (10.8%) than other groups (P = 0.001). Overall, the need for psychiatric consultation was reported in 13.8% of cases and the mortality rate was 6.6% (n = 98/1492) for each patient during the index admission across the study period. With respect to different age groups, the need for psychiatric consultation (18.1%) and mortality (16.5%) was significantly higher in the age group of 45–54 years (P = 0.001 for both) than the other age groups.

Table 3 demonstrates the biochemical investigation and level of BAC and ethanol according to the age groups. Older patients ( ≥ 55 years) had significantly elevated blood glucose levels (7.06 [5.3–10.2]), HbA1c (6.8 [5.9–7.6]), blood urea (4.5 [3.5–6.2]), creatinine (82.0 [70.0–96.0]), and lower median hemoglobin levels (14.4 [12.9–15.4]) as compared to other groups (P = 0.001 for all). The median levels of alkaline phosphatase (82.0 [64.0–104.5]), alanine aminotransferase (44.0 [26.0–70.8]), aspartate aminotransferase (47.0 [27.8–91.3]), and total bilirubin (8.3 [5.0–15.1]) levels were highest in the age group of 35–44 years than the other age groups (P = 0.001).

**Figure 1a.**
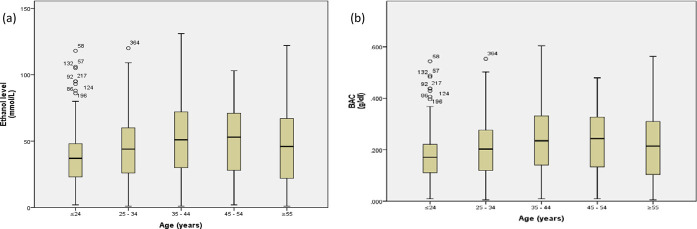
and b. Box plots comparing ethanol levels and BAC among different age groups

**Figure 2.**
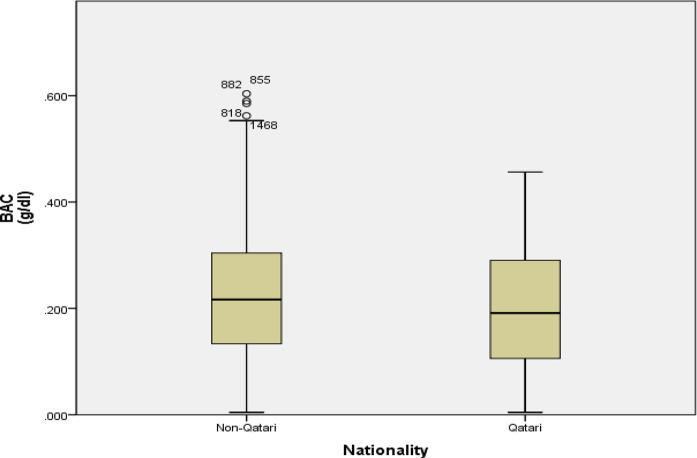
Box plot comparing BAC between Qataris and non-Qataris

The median levels of ethanol were the highest in the age groups of 35–44 years (51.0 [30.0–72.0]) and 45–54 years (53.0 [28.0–71.5]) as compared to the other age groups (P = 0.001). The same pattern was also found for BAC with the highest levels being in the age groups of 35–44 years (0.24 [0.14–0.33]) and 45–54 years (0.24 [0.13–0.33]) (Figure 1a and b). The lowest medians of ethanol and BAC levels were found in the age group of < 25 years (37.0 [23.0–48.0] and 0.17 [0.11–0.22], respectively]. Figure 2 shows that among alcohol consumers, non-Qatari and Qatari patients had comparable BAC levels (0.22 ± 0.12 vs. 0.20 ± 0.11).

Tables 4 and 5 show the demographic characteristics and outcomes of participants according to the age groups between Qataris and non-Qataris, respectively. In the Qatari sub-group (29% of the cohort), the study age groups were comparable for gender. Patients with advanced age ( ≥ 55 years) were more likely to have recurrent admissions mostly not related to trauma (P = 0.001), whereas younger patients ( < 25 years) were frequently admitted because of traumatic injuries. Patients in the age group of 45–54 years had a higher mortality rate than the other groups.

Among non-Qataris (71% of the cohort), there were more frequent trauma-related admissions in the age group of 25–34 years (P = 0.001). With respect to different age groups, the rate of mortality among non-Qataris (10.9%) was significantly higher in the older age group ( ≥ 55 years, P = 0.001).

## Discussion

This study describes the socioeconomic characteristics, comorbidities, and hospitalization/ED visit patterns attributable to alcohol consumption based on age from a national referral hospital in the State of Qatar. In 2014, the total population of Qatar was 2,123,160 (15% Qataris and 85% non-Qataris); 70% of the population was in the age range 25–54 years and male predominated (80%) as compared to female (20%).^12^ The majority of non-Qataris were from South-East Asia. A previous study showed that the average number of ED visits exceeds 1000 patients per day at the HGH.^13^ Notably, the ED at HGH performs alcohol screening for all suspected cases and anecdotal reports suggested that there is an increasing number of admissions that are linked to alcohol consumption, which could pose a burden on the ED and outpatient clinics.

In our study cohort, 71% were non-Qataris and the rest (29%) were Qataris. The mortality was higher among Qataris (16.4%) as compared to 2.5% among the non-Qataris. This difference in the mortality rate can be explained in part by the fact that 53% of the Qataris in this study had underlying comorbidities and were older (above the age of 45 years), whereas 84% of the affected non-Qataris were younger in age. An earlier study from our center showed that 38% of the screened patients were positive for blood alcohol indicating the magnitude of the problem, which perhaps needs further exploration.^10^ Therefore, this study has focused on alcohol-related ED visits based on age distribution with several key findings. The pattern of hospital admission, sociodemographic status, presence of comorbidities, laboratory investigations, and mortality showed specific age-related distribution. Particularly, young adults were more likely to have ED visits due to trauma, whereas older patients mostly had hospital admissions related to underlying comorbidities. Patients aged 35–44 years had a significantly elevated liver function profile, whereas blood investigations indicating diabetes and kidney disease were more prominent in the older patients ( ≥ 55 years).

Our findings are consistent with the population's gender distribution in Qatar, which showed that three-fourths of the population was males.^14^ However, a higher male predominance (95.6%) among alcoholics could be explained by the sociocultural characteristics, which are unique in the Gulf region as opposed to the Western world. Furthermore, our findings corroborate with earlier studies from other countries that reported a higher frequency of alcohol-related ED visits among males which ranges between 51% and 72%.^4,15–17^ Notably, the pattern of alcohol consumption may vary based on personal habits, culture, and country. According to the study findings and based on data for patients who were seen and screened in the ED; higher alcohol levels were identified in the age groups of 35–44 and 45–54 years. This could be explained by the fact that most expatriates in Qatar are mainly comprised of the working young-age group and often belong to countries where alcohol consumption is permissible. Therefore, a strong disciplinary action against the driver with evidence of alcoholism could be effective. The restricted availability of alcohol in the Middle Eastern countries may lead to the production of home-brewed alcohol that may have a higher ethanol content than the licensed alcoholic beverages. Data from Europe showed that a heavy alcohol consumption is more common in adolescents and young adults.^18^ Some studies have reported a decline in the amount of alcohol consumption with increasing age, but the habit of alcohol consumption persisted among older individuals than the younger age population.^11^ Also, a previous study reported that the age group of 25–34 years had higher BAC values, which are relatively lower than the observed values of this study.^10^ It is noteworthy that the period after 2012 has shown a more influx of expatriates in the country with intensification for the 2022 FIFA World Cup Football preparations in Qatar.

In this study, patients in the older age group were more likely to have recurrent hospital admission, whereas young adults had hospitalization due to traumatic injuries. Similarly, Savola et al.,^19^ showed that young individuals and working-age adults were more likely to have traumatic injuries secondary to heavy alcohol consumption. As in this study, the drinking issue appears to be linked to the working-age adults (25–34 and 35–44 years). However, older adults are more likely to have frequent hospitalization due to alcohol-related complications and associated comorbidities.^20^

The ACCORD Trial showed an association between moderate alcohol intake and the risk of hypertension in patients with diabetes mellitus and cardiovascular risk factors.^21^ Our findings also showed a higher predilection of comorbidities such as diabetes mellitus, hypertension, liver dysfunction, and CAD among older patients ( ≥ 55 years). In our cohort, thiamine therapy was prescribed in more than half of the cases across the different age groups, whereas benzodiazepines were given to more than a quarter of cases. There are few published studies regarding alcohol consumption in the Arab Middle Eastern region.^7^ This is possibly attributed to the legal issues and restrictions regarding alcohol consumption; therefore, investigators may find it difficult to objectively study the prevalence and patterns of alcohol consumption across the Arab Middle Eastern countries. Furthermore, the stigma of alcohol abuse may also discourage research in this field.^22,23^ A previous study^10^ speculated that there might be more cases of alcohol consumption that remain unidentified because of the lack of protocolized alcohol screening in different hospitals. Some studies from the Arab Middle Eastern region have suggested that the implementation of screening and brief intervention protocol for alcohol abuse among patients visiting the ED is beneficial to prevent harmful consequences.^24,25^ A mandatory screening protocol for alcohol consumption in trauma patients has been implemented in the HGH since 2016, which may be beneficial to assess the association of alcohol use with traumatic injuries.^26^

One of the strengths of our study is that it is one of few studies on alcohol consumption in Qatar that could be used as a basis for implementing screening protocols for the objective measurement of BAC and ethanol in ED of all the hospitals across Qatar as well as identifying the high-risk population. Therefore, public awareness campaigns, occupational seminars, policies, and regulations should be implemented in countries dealing with the problem of alcohol abuse.

One of the key limitations of our study is the retrospective design that has selection bias and missing information, which impacted the generalizability of our findings. This study lacks information regarding the number of patients screened for alcohol across HMC; therefore, our results are not representative of the whole population. However, this does not diminish the importance of our findings, as this study has a sizable number of patients who were screened positive for alcohol in the ED during the study period. Another limitation of this study is the lack of information regarding what prompted the BAC screening test and the underlying primary reason for the patients being seen and admitted to the ED at that time.

## Conclusion

This study highlighted the pattern of age and clinico-epidemiological status of patients with alcohol-attributable hospital admissions. Our study showed that alcohol consumption was higher among the working-age group (34–54 years) of the population and mostly among expatriates, reflecting the country's general population demographics. This result shows that alcohol consumption poses a healthcare burden irrespective of partial ban on alcohol. Moreover, alcohol-related ED visits were more frequent in the age group 35–54 years among Qataris, whereas the age range in non-Qataris was relatively younger (25–44 years). A standard protocol needs to be established for alcohol screening in the ED to monitor the demographic pattern that may help in developing specific interventions. Therefore, national monitoring systems and protocols need to be established to keep track of alcohol consumption, implement suitable interventions to reduce alcohol-related injuries, and public awareness campaigns should target high-risk populations. Further studies are needed to investigate the pattern of alcohol consumption, which may help plan health resources and prevention of alcohol-related problems.

### Acknowledgments

We thank Prof Egon Toft, Prof Michail Nomikos, Mr Tawanda Chivese, (Faculty of Medicine, Qatar University) and Dr Hassan Al-Thani (Trauma Surgery, HMC) for their support and mentorship.

### Ethical issues

The Institutional Review Board at Hamad Medical Corporation (MRC15447/15) and Ethics Committee of Qatar University (QU-IRB 1098-E/19) approved this observational study.

### Sponsor, funding

 None

### Conflict of interest

No conflict of interest to declare.

## Figures and Tables

**Table 1:** Comparison of demographic characteristics of participants according to the age groups

Variable	Overall n = 1506	< 25 yrs (13.6%)	25–34 yrs (30.9%)	35–44 yrs (28.4%)	45–54 yrs (17.4%)	≥ 55 yrs (9.7%)	P-value

**Gender (%)**							

Females	66/1506 (4.4)	8/205 (3.9)	21/465 (4.5)	26/428 (6.1)	10/262 (3.8)	1/146 (0.7)	0.091

Males	1440/1506 (95.6)	197/205 (96.1)	444/465 (95.5)	402/428 (93.9)	252/262 (96.2)	145/146 (99.3)	

**Nationality (%)**							

Qatari	435/1506 (28.9)	34/205 (16.6)	69/465 (14.8)	102/428 (23.8)	141/262 (53.8)	89/146 (61.0)	0.001

Non-Qatari	1071/1506 (71.1)	171/205 (83.4)	396/465 (85.2)	326/428 (76.2)	121/262 (46.2)	57/146 (39.0)	

**Patterns of admission (%)**							

Recurrent	344/561 (61.3)	23/61 (37.7)	53/ 126(42.1)	107/160 (66.9)	91/124 (73.4)	70/90 (77.8)	0.001 for all

Different	217/561 (38.7)	38/61 (62.3)	73/126 (57.9)	53/160 (33.1)	33/124 (26.6)	20/90 (22.2)	

**Previous hospital admission (%)**							

Trauma	72/502 (14.3)	11/49 (22.4)	18/93 (19.4)	20/149 (13.4)	18/124 (14.5)	5/87 (5.7)	0.04 for all

Nontrauma	430/502 (85.7)	38/49 (77.6)	75/93 (80.6)	129/149 (86.6)	106/124 (85.5)	82/87 (94.3)	

**Previous ED visit (%)**							

Trauma	55/575 (9.6)	9/55 (16.4)	15/119 (12.6)	16/172 (9.3)	10/138 (7.2)	5/91 (5.5)	0.14 for all

Nontrauma	520/575 (90.4)	46/55 (83.6)	104/119 (87.4)	156/172 (90.7)	128/138 (92.8)	8691 (94.5)	

**Marital status (%)**							

Single	174/593 (29.3)	64/76 (84.2)	63/105 (60.0)	28/168 (16.7)	14/158 (8.9)	5/86 (5.8)	0.001 for all

Married	396/593 (66.8)	11/76 (14.5)	41/105 (39.0)	126/168 (75.0)	138/158 (87.3)	80/86 (93.0)	

Divorced	22/593 (3.7)	0/76 (0.0)	1/105 (1.0)	14/168 (8.3)	6/158 (3.8)	1/86 (1.2)	

Widowed	1/593 (0.2)	1/76 (1.3)	0/105 (0.0)	0/168 (0.0)	0/158 (0.0)	0/86 (0.0)	

**Employment (%)**							

Not working	47/496 (9.5)	13/61 (21.3)	17/119 (14.3)	7/141 (5.0)	6/96 (6.3)	4/79 (5.1)	0.001 for all

Working	390/496 (78.6)	45/61 (73.8)	99/119 (83.2)	127/141 (90.1)	61/96 (63.5)	58/79 (73.4)	

Retired	59/496 (3.0)	3/61 (4.9)	3/119 (3.0)	7/141 (5.0)	29/96 (30.2)	17/79 (21.5)	

**Year of admission (%)**							

2013	477/1506 (31.7)	54/205 (26.3)	143/465 (30.8)	133/428 (31.1)	82/262 (31.3)	65/146 (44.5)	0.013 for all

2014	836/1506 (55.5)	131/205 (63.9)	252/465 (54.2)	241/428 (56.3)	145–262 (55.3)	67/146 (45.9)	

2015*	193/1506 (12.8)	20/205 (9.8)	70/465 (15.1)	54/428 (12.6)	35/262 (13.4)	14/146 (9.6)	


*Data from January to March, the percentage is given in bracket, yrs = years old

**Table 2:** Comorbidities, admitting services, and mortality according to the age groups

Variable	Overall n = 1506	< 25 yrs (13.6%)	25–34 yrs (30.9%)	35–44 yrs (28.4%)	45–54 yrs (17.4%)	≥ 55 yrs (9.7%)	P-value

**Comorbidities (%)**							

Hypertension	264/972 (27.2)	1/137 (0.7)	15/292 (5.1)	76/279 (27.2)	90/157 (57.3)	82/107 (76.6)	0.001

Diabetes mellitus	215/950 (22.6)	0/137 (0.0)	23/301 (7.6)	50/260 (19.2)	75/157 (47.8)	67/95 (70.5)	0.001

Liver disease	98/755 (13.0)	0/122 (0.0)	1/259 (0.4)	38/218 (17.4)	33/95 (34.7)	26/61 (42.6)	0.001

IHD	73/759 (9.6)	0/127 (0.0)	5/264 (1.9)	5/199 (2.5)	31/103 (30.1)	32/66 (48.5)	0.001

Cardiac arrest	18/703 (2.6)	1/128 (0.8)	4/260 (1.5)	2/192 (1.0)	7/82 (8.5)	4/41 (9.8)	0.001

Cardiac arrhythmia	24/696 (3.4)	1/123 (0.8)	1/260 (0.4)	2/189 (1.1)	8/83 (9.6)	12/41 (29.3)	0.001

Congestive heart failure	16/705 (2.3)	0/128 (0.0)	1/259 (0.4)	1/192 (0.5)	6/82 (7.3)	8/44 (18.2)	0.001

Atrial fibrillation	68/75 (90.7)	3/3 (100)	8/10 (80.0)	19/20 (95.0)	20/23 (87.0)	18/19 (94.7)	0.58

**Treatment (%)**							

Thiamine (Vit B1)	750/1314 (57.1)	99/185 (53.5)	250/419 (59.7)	213/370 (57.6)	120/209 (57.4)	68/131 (51.9)	0.466

Benzodiazepine	337/1309 (25.7)	46/183 (25.1)	95/420 (22.6)	93/368 (25.3)	64/207 (30.9)	39/131 (29.8)	0.182

**Admitting services (%)**							

Emergency short stay	1124/1312 (85.7)	145/177 (81.9)	335/407 (82.3)	329/372 (88.4)	208/232 (89.7)	107/124 (86.3)	0.025

Trauma room	279/1376 (20.3)	50/188 (26.6)	121/437 (27.7)	61/376 (16.2)	26/250 (10.4)	21/125 (16.8)	0.001

Medical/surgical ward	140/1276 (11.0)	20/180 (11.1)	37/405 (9.1)	40/344 (11.6)	28/230 (12.2)	15/12.8 (12.8)	0.680

Cardiac ward	24/1263 (1.9)	0/175 (0.0)	0/403 (0.0)	6/344 (1.7)	6/230 (2.6)	12/111 (10.8)	0.001

Trauma ICU	87/1319 (6.6)	18/180 (10.0)	36/409 (8.2)	16/361 (4.4)	10/247 (4.0)	7/122 (5.7)	0.017

Medical ICU	29/1276 (2.3)	2/178 (1.1)	3/404 (0.7)	10/340 (2.9)	6/238 (2.5)	8/116 (6.9)	0.002

Surgical ICU	20/1282 (1.6)	3/178 (1.7)	2/404 (0.5)	7/341 (2.1)	6/243 (2.5)	2/116 (1.7)	0.301

Cardiac ICU	7/1252 (0.6)	1/176 (0.6)	1/404 (0.2)	1/339 (0.3)	1/232 (0.4)	3/101 (3.0)	0.019

**Psychiatric consultation (%)**	150/1087 (13.8)	17/158 (10.8)	35/354 (9.9)	46/279 (16.5)	36/199 (18.1)	16/97 (16.5)	0.025

**Mortality (%)**	98/1492 (6.6)	2/200 (1.0)	6/463 (1.3)	27/425 (6.4)	43/260 (16.5)	20/144 (13.9)	0.001


IHD: ischemic heart disease, ICU: intensive care unit, the percentage is given in bracket

**Table 3:** Biochemical tests and level of BAC and ethanol according to the age groups

Variable	Overall n = 1506 (100%)	< 25 yrs n = 205 (13.6%)	25–34 yrs n = 465 (30.9%)	35–44 yrs n = 428 (28.4%)	45–54 yrs n = 262 (17.4%)	≥ 55 yrs n = 146 (9.7%)	P-value

Hemoglobin, g/dl (median (IQR))	15.1 (13.9–16.1)	15.2 (14.2–16.0)	15.3 (14.2–16.3)	15.3 (14.0–16.3)	14.7 (13.4–15.8)	14.4 (12.9–15.4)	0.001

HbA1c, % (median (IQR))	6.1 (5.4–7.8)	5.2 (5.2–5.2)	5.20 (4.9–5.6)	6.0 (5.4–7.5)	6.6 (5.5–9.0)	6.8 (5.9–7.6)	0.001

Glucose, mmol/L [1st reading only] (median (IQR))	6.2 (5.4–7.4)	6.1 (5.5–6.9)	6.08 (5.3–7.0)	6.2 (5.4–7.5)	6.06 (5.3–7.8)	7.06 (5.3–10.2)	0.001

Creatinine μmol/L (median (IQR))	77.0 (66.0–90.0)	77.0 (66.5–90.0)	79.0 (69.0–93.0)	75.0 (65.0–86.0)	73.0 (62.3–86.0)	82.0 (70.0–96.0)	0.001

BUN, mmol/L (median (IQR))	3.6 (2.8–4.5)	3.4 (2.7–4.2)	3.5 (2.8–4.3)	3.6 (2.8–4.5)	3.55 (2.6–4.6)	4.5 (3.5–6.2)	0.000

Albumin, g/L [ALB] (median (IQR))	41.0 (37.0–44.0)	42.0 (38.0–46.0)	42.0 (38.0–46.0)	41.0 (37.0–44.0)	41.0 (35.0–44.0)	40.0 (36.0–44.0)	0.020

ALT; IU/L (median (IQR))	35.0 (23.0–46.0)	29.0 (19.0–46.0)	35.0 (22.0–56.8)	44.0 (26.0–70.8)	39.0 (24.0–67.5)	30.0 (22.0–46.0)	0.000

AST (median (IQR))	38.0 (25.0–76.5)	29.0 (21.0–47.3)	32.0 (22.0–52.0)	47.0 (27.8–91.3)	47.0 (27.0–94.0)	35.0 (24.3–56.5)	0.000

Total Bilirubin, μmol/L (median (IQR))	7 (4.9–12.0)	6.9 (4.8–10.9)	6.7 (4.8–10.3)	8.3 (5.0–15.1)	7.5 (4.8–13.0)	6.6 (4.4–10.9)	0.001

ALP, IU/L (median (IQR))	76.0 (60.0–96.0)	72.5 (57.0–95.2)	69.0 (55.0–84.5)	82.0 (64.0–104.5)	79.0 (63.3–98.8)	73.0 (58.0–91.0)	0.000

GGT, U/L (median (IQR))	59.0 (33.0–177.5)	61.0 (61.0–61.0)	55.0 (31.5–83.8)	98.5 (29.5–300.0)	54.0 (22.0–392.0)	86.0 (33.0–437.0)	0.880

Lactic acid, mmol/L (median (IQR))	2.9 (2.3–3.9)	2.88 (2.3–4.2)	2.7 (2.2–3.5)	2.9 (2.3–4.2)	3.1 (2.4–4.3)	3.1 (2.1–4.6)	0.686

ETOH, mmol/L (median (IQR))	46.0 (26.0–65.0)	37.0 (23.0–48.0)	44.0 (26.0–60.0)	51.0 (30.0–72.0)	53.0 (28.0–71.5)	46.0 (21.3–67.1)	0.001

BAC, g/dl (median (IQR))	0.21 (0.12–0.29)	0.17 (0.11–0.22)	0.20 (0.12–0.28)	0.24 (0.14–0.33)	0.24 (0.13–0.33)	0.21 (0.10–0.31)	0.001


Gamma-glutamyl transferase (GGT); alkaline phosphatase (ALP); alanine aminotransferase (ALT); Aspartate aminotransferase (AST); Blood alcohol concentration (BAC). Blood urea nitrogen (BUN); Ethanol levels (ETOH)

**Table 4:** Demographic characteristics and outcome of participants according to the age groups among Qataris patients

Variable	Overall	< 25 yrs	25–34 yrs	35–44 yrs	45–54 yrs	≥ 55 yrs	P-value

**Gender (%)**							

Females	14/435 (3.2)	1/34 (2.9)	2/69 (2.9)	4/102 (3.9)	7/141 (5.0)	0/89 (0.0)	0.33 for all

Males	421/435 (96.8)	33/34 (97.1)	67/69 (97.1)	98/102 (96.1)	134/141 (95.0)	89/89 (100)	

Patterns of admission (%)							

Recurrent	193/303 (63.7)	5/23 (21.7)	23/55 (41.8)	46/67 (68.7)	69/94 (73.4)	50/64 (78.1)	0.001 for all

Different	110/303 (36.3)	18/23 (78.3)	32/55 (58.2)	21/67 (31.3)	25/94 (26.6)	14/64 (21.9)	

Trauma-related	50/302 (16.6)	8/23 (34.8)	6/42 (14.3)	14/71 (19.7)	18/103 (17.5)	4/63 (6.3)	0.02 for all

Non-trauma	252/302 (83.4)	15/23 (65.2)	36/42 (85.7)	57/71 (80.3)	85/103 (82.5)	59/63 (93.7)	

**Marital status (%)**							

Single	85/353 (24.1)	24/28 (85.7)	35/50 (70.0)	15/92 (16.3)	8/125 (6.4)	3/58 (5.2)	0.001 for all

Married	248/353 (70.3)	3/28 (10.7)	14/50 (28.0)	66/92 (71.7)	111/125 (88.8)	54/58 (93.1)	

Divorced	19/353 (5.4)	0/28 (0.0)	1/50 (2.0)	11/92 (12.0)	6/125 (4.8)	1/58 (1.7)	

Widowed	1/353 (0.3)	1/28 (3.6)	0/50 (0.0)	0/92 (0.0)	0/125 (0.0)	0/58 (0.0)	

**Employment (%)**							

Not working	35/236 (14.8)	7/21 (33.3)	11/41 (26.8)	7/57 (12.3)	6/63 (9.5)	4/54 (7.4)	0.001 for all

Working	145/236 (61.4)	11/21 (52.4)	27/41 (65.9)	44/57 (77.2)	28/63 (44.4)	35/54 (64.8)	

Retired	56/236 (23.7)	3/21 (14.3)	3/41 (7.3)	6/57 (10.5)	29/63 (46.0)	15/54 (27.8)	

Mortality (%)	71/432 (16.4)	2/34 (5.9)	0/69 (0.0)	20/100 (20.0)	35/140 (25.0)	14/89 (15.7)	0.001


**Table 5:** Demographic characteristics and outcome of participants according to the age groups among non-Qatari patients

Variable	Overall	< 25 yrs	25–34 yrs	35–44 yrs	45–54 yrs	≥ 55 yrs	P-value

**Gender (%)**							

Females	52/1071 (4.9)	7/171 (4.1)	19/396 (4.8)	22/326 (6.7)	3/121 (2.5)	1/57 (1.8)	0.24 for all

Males	1019/1071 (95.1)	164/171 (95.9)	377/396 (95.2)	304/326 (93.3)	118/121 (97.5)	56/57 (98.2)	

**Patterns of admission (%)**							

Recurrent	151/258 (58.5)	18/38 (47.4)	30/71 (42.3)	61/93 (65.6)	22/30 (73.3)	20/26 (76.9)	0.001 for all

Different	107/258 (41.5)	20/38 (52.6)	41/71 (57.7)	32/93 (34.4)	8/30 (26.7)	6/26 (23.1)	

Trauma-related	22/200 (11.0)	3/26 (11.5)	12/51 (23.5)	6/78 (7.7)	0/21 (0.0)	1/24 (4.2)	0.01 for all

Nontrauma	178/200 (89.0)	23/26 (88.5)	39/51 (76.5)	72/78 (92.3)	21/21 (100)	23/24 (95.8)	

**Marital status (%)**							

Single	89/240 (37.1)	40/48 (83.3)	28/55 (50.9)	13/76 (17.1)	6/33 (18.2)	2/28 (7.1)	0.001 for all

Married	148/240 (61.7)	8/48 (16.7)	27/55 (49.1)	60/76 (78.9)	27/33 (81.8)	26/28 (92.9)	

Divorced	3/240 (1.3)	0/48 (0.0)	0/55 (0.0)	3/76 (3.9)	0/33 (0.0)	0/28 (0.0)	

**Employment (%)**							

Not working	12/260 (4.6)	6/40 (15.0)	6/78 (7.7)	0/84 (0.0)	0/33 (0.0)	0/25 (0.0)	0.001 for all

Working	245/260 (94.2)	34/40 (85.0)	72/78 (92.3)	83/84 (98.8)	33/33 (100)	23/25 (92.0)	

Retired	3/260 (1.2)	0/40 (0.0)	0/78 (0.0)	1/84 (1.2)	0/33 (0.0)	2/25 (8.0)	

Mortality (%)	27/1060 (2.5)	0/166 (0.0)	6/394 (1.5)	7/325 (2.2)	8/120 (6.7)	6/55 (10.9)	0.001

